# Pre-Ball-Milled Boron Nitride for the Preparation of Boron Nitride/Polyetherimide Nanocomposite Film with Enhanced Breakdown Strength and Mechanical Properties for Thermal Management

**DOI:** 10.3390/nano12193473

**Published:** 2022-10-04

**Authors:** Ruiyi Li, Xiao Yang, Jian Li, Ding Liu, Lixin Zhang, Haisheng Chen, Xinghua Zheng, Ting Zhang

**Affiliations:** 1 Nanjing Institute of Future Energy System, Nanjing 211135, China; 2 Institute of Engineering Thermophysics, Chinese Academy of Sciences, Beijing 100190, China; 3 University of Chinese Academy of Sciences, Beijing 100049, China; 4 Innovation Academy for Light-Duty Gas Turbine, Chinese Academy of Sciences, Beijing 100190, China

**Keywords:** polymer composites, boron nitride nanosheets, thermal conductivity, breakdown strength, mechanical properties

## Abstract

Modern electronics not only require the thermal management ability of polymer packaging materials but also need anti-voltage and mechanical properties. Boron nitride nanosheets (BNNS), an ideal thermally conductive and high withstand voltage (800 kV/mm) filler, can meet application needs, but the complex and low-yield process limits their large-scale fabrication. Herein, in this work, we prepare sucrose-assisted ball-milled BN(SABM-BN)/polyetherimide (PEI) composite films by a casting-hot pressing method. SABM-BN, as a pre-ball-milled filler, contains BNNS and BN thick sheets. We mainly investigated the thermal conductivity (TC), breakdown strength, and mechanical properties of composites. After pre-ball milling, the in-plane TC of the composite film is reduced. It decreases from 2.69 to 2.31 W/mK for BN/PEI composite film at 30 wt% content; however, the through-plane TC of composites is improved, and the breakdown strength and tensile strength of the composite film reach the maximum of 54.6 kV/mm and 102.7 MPa at 5 wt% content, respectively. Moreover, the composite film is used as a flexible circuit substrate, and the working surface temperature is 20 ℃, which is lower than that of pure PEI film. This study provides an effective strategy for polymer composites for electronic packaging.

## 1. Introduction

As electronic devices become increasingly miniaturized and intelligent, their energy density rises sharply and can be up to 300 W/cm^2^ [1,2,3]. Local “hot spots” will have an irreversible impact on the life and stable use of electronic components. Because of their packaging and thermal management capabilities, thermally conductive polymer films are increasingly attracting attention, with the expectation of expanding their applications in flexible display screens [4], circuit substrates [5], and highly integrated insulated gate bipolar transistors (IGBT) [6].

Common insulating polymer films in electronic devices include polyimide (PI) [7], epoxy (EP) resin [8], and polyvinylidene fluoride (PVDF) [9]. However, the intrinsic thermal conductivity of this type of polymer is low, <0.2 W/mK [10]. It is often necessary to compound thermally conductive fillers to prepare thermal management materials (TMMs). Nevertheless, modern electronics also impose anti-voltage and mechanical performance requirements on TMMs to ensure low electrical failure rates and high reliability [11,12].

Boron nitride nanosheets (BNNS) have a two-dimensional hexagonal structure similar to graphene, high thermal conductivity (600 W/mK) [13,14,15], and ultra-high breakdown strength (800 kV/mm) [16], which are ideal choices for thermal management and anti-voltage polymer composites. Song et al. [17] incorporated 2 wt% BNNS into PVDF. The thermal conductivity was improved to 0.28 W/mK, and the breakdown strength increased from 366 kV/mm of pure film to 450 kV/mm. The common preparation process of BNNS includes ball milling [18], chemical exfoliation [19], and tip sonicate [20]. Note that these methods have so far been too complex, inefficient (~10 wt% yield), and very unsuitable for large-scale manufacturing. Still, the corresponding unexfoliated BN is more likely to form heat conduction networks than small-sized BNNS [21,22,23]. The size difference between bulk BN and BNNS may bring related synergies. Gu et al. [24] employed hybrid fillers of micrometer BN and nanometer BN with polyphenylene sulfide to hot-press into the highly thermal conductive composites. It was found that hybrid fillers were more beneficial in improving the thermal conductivity of composites compared to single-size fillers. These advantages are ignored, and the bulk BN is wasted by blindly pursuing BNNS. In addition, the functionalization of fillers, such as hydroxyl [25] or amino groups [26], should also be considered in the exfoliated process to increase the affinity with the polymer matrix and improve the mechanical properties [27,28,29].

Consequently, in this work, we use commercially available and cheap sucrose as grinding for exfoliating and modifying BN. Furthermore, polyetherimide (PEI), a mature and commercial PI, is selected as the matrix. The thermal conductivity, breakdown strength, and mechanical properties of composite films before and after pre-ball milling BN are emphatically compared. The film prepared from BN by sucrose-assisted ball milling is named SABM-BN/PEI composite film, and the untreated version is named BN/PEI composite film. As a result, the in-plane thermal conductivity of the SABM-BN/PEI composite film decreases to a certain extent compared with the BN/PEI composite film. However, the through-plane thermal conductivity of the SABM-BN/PEI composite film is improved. The breakdown strength of the SABM-BN/PEI composite film is higher than that of the BN/PEI composite film in the entire content range. For mechanical properties, the tensile strength of the composite film shows a similar trend to the breakdown strength. This improvement is attributed to the interface interaction. Verifying the thermal management capability of the SABM-BN/PEI composite film, the film surface uses screen printing to prepare a flexible circuit. The surface had a lower working temperature compared with commercial paper and PEI. Compared with previous work, this paper does not carry out subsequent separation after ball milling. The composite film prepared by combining the advantages of BNNS and BN has anti-voltage, excellent mechanical properties, and thermal management ability. The above shows that the prepared SABM-BN/PEI composite film has great potential to be used in electronics packaging and unique applications.

## 2. Materials and Methods

### 2.1. Materials

Polyetherimide powder (PEI, Ultem 1000) was purchased from SABIC Innovative Plastics Co., Ltd. (Shanghai, China). Boron nitride platelets (BN, ~20 μm) were supplied by Dandong Rijin Science and Technology Co., Ltd. (Dandong, China). *N,N*-dimethyl formamide (DMF) and sugar were provided by Shanghai Aladdin Biochemical Technology Co., Ltd. (Shanghai, China).

### 2.2. Preparation of Sugar-Assisted Ball-Milled BN Powder

In the dry ball milling process [30,31], BN (1 g), sugar (5 g), and zirconia balls (60 g) were loaded into a 500 mL grinding jar. The zirconia balls were divided into three kinds with diameters of 10 mm, 5 mm, and 1 mm in a weight ratio of 2:5:3. The mixture was ball milled at 500 rpm for 24 h using a planetary ball mill (YXQM-2L, MITR, China). Then D.I. water (200 mL) was poured into the mixture and stirred to speed up the dissolution of the sucrose. Finally, the suspension was vacuum filtered through an aqueous filter membrane (pore size: 0.22 μm) and washed several times to remove residual sucrose, followed by vacuum drying to obtain sugar-assisted ball-milled BN (SABM-BN). The SABM-BN contained the BN thick sheets (BNTS) and BN nanosheets (BNNS).

### 2.3. Preparation of PEI Composite Films

**Figure 1:**
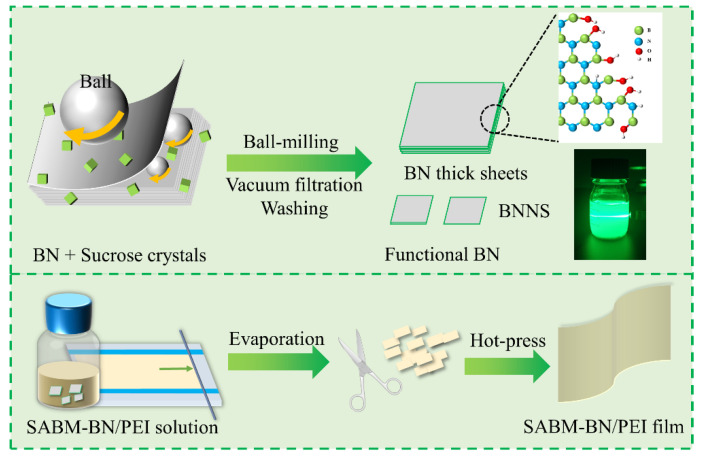
Schematic diagram illustrating the preparation of the SABM-BN and composite film.

The preparation process of the BN/PEI and SABM-BN/PEI composite films is shown in Figure 1. First, BN or SABM-BN was ultrasonically dispersed in DMF (40 mL). Next, PEI powder (10 g) was added to the aforementioned DMF to dissolve. It was stirred at 80 ℃ for 4 h and sonicated to obtain a homogeneous composite solution. Then, the solution was cast on a clean glass plate. The glass plate was placed in an oven (160 ℃) to remove the DMF. Subsequently, the film was peeled off the glass plate and shredded. The cut samples were placed between two layers of stainless-steel plates, and the mold release paper was Teflon fiber cloth. Finally, it was hot-pressed at 1 MPa, 270 ℃ for 10 min using a hot-pressing machine (6170B, BOLON, Dongguan, China) to obtain PEI composite films. Filler content was set at 0, 5, 10, 20, and 30 wt%.

### 2.4. Characterization

The Fourier transform infrared (FT-IR, Tensor 27, Bruker Nano Gmbh, Berlin, Germany) spectrum of the BN, SABM-BN, and sugar were collected using pressed KBr pellets. The filler and film cross-section morphologies were observed using a field emission scanning electronic microscope (FESEM, Regulus 8100, Hitachi, Tokyo, Japan). Before testing, the two samples were sprayed with a thin layer of gold for 90 s and 120 s. The differential scanning calorimetry (DSC, 204F1, Netzsch, Zelb, Germany) was performed to investigate the glass transition temperature (Tg). The films were heated to 300 ℃ at a 10 K/min heating rate under N_2_ atmosphere. The mechanical properties of the composite films were measured on an electronic universal testing machine (105D-TS, Wance, Shenzhen, China) with a tensile rate of 1 mm/min. The breakdown strength was obtained using a withstand voltage tester (CD9917-AX, Changsheng, Nanjing, China) at a ramping voltage rate of 1 kV/s. A thermal constant analyzer (TPS2500s, Hot Disk AB, Västerås, Sweden) was used to determine the thermal conductivity of the composite film at room temperature. The heat resistance of the composite films was recorded using a thermal gravimetric analyzer (TG, 209F3, Netzsch, Zelb, Germany) from room temperature to 800 ℃ at N_2_ atmosphere. To investigate the heat dissipation of the composite films in flexible electronic devices, first, silver circuits were screen-printed on the surface of the samples, followed dried. Next, a constant 12 V voltage was applied to the sample surface circuit, and the thermal distribution on the surface was simultaneously recorded using an infrared thermal imager (PS400, Guide, Wuhan, China).

## 3. Results and Discussion

**Figure 2:**
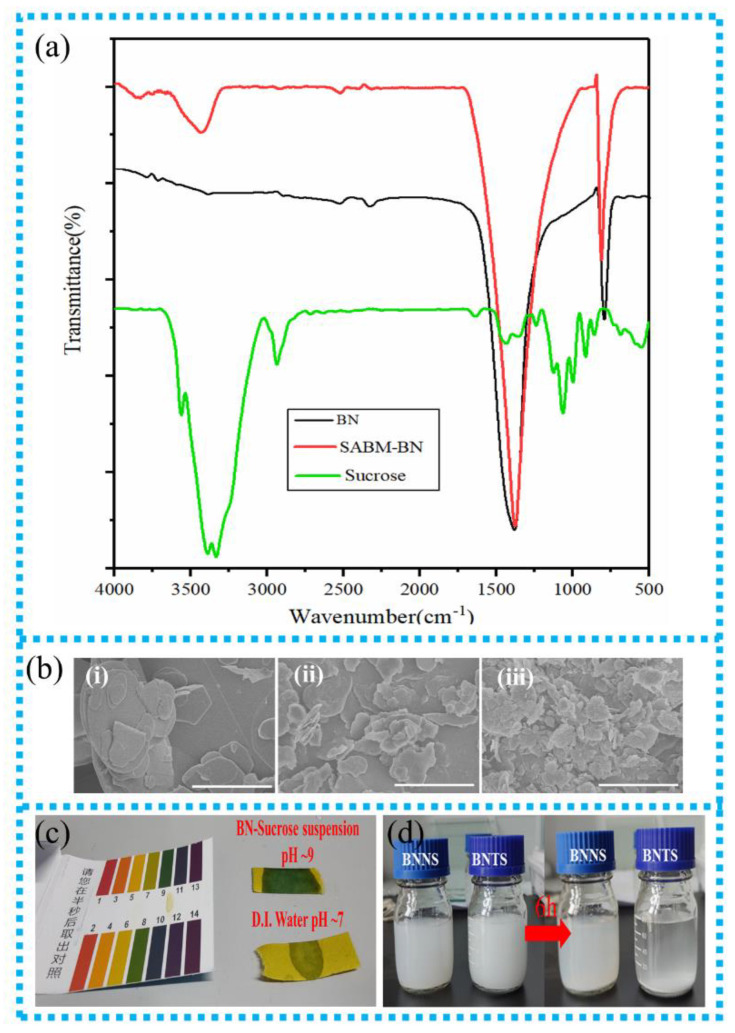
(**a**) FT-IR spectra of BN, sucrose, and SABM-BN. (**b**) SEM images of (**i**) BN, (**ii**) BNTS, and (**iii**) BNNS, respectively (scale bar: 10 μm). (**c**) Detection pH of BN-Sucrose suspension and D.I. water. (**d**) Stability of BNNS and BNTS (~1 mg/mL) aqueous solution for 6 h.

Figure 1 shows the preparation process of the SABM-BN/PEI composite film. First, sucrose and BN are ball milled to obtain the SABM-BN, followed by casting and hot pressing to prepare the composite films. It is worth mentioning that, since the PEI solution easily absorbs moisture and causes irreversible phase transition during the casting process, it is necessary to further hot-press to obtain a dense composite film. As shown in Figure 2a, the peaks at 1375 cm^−1^ and 809 cm^−1^ on BN and SABM-BN correspond to the stretching and bending vibrations of B–N [32,33], respectively. It is found that some characteristic peaks of sucrose do not appear on the SABM-BN curve, which also indicates that the sucrose is completely removed during washing. In addition, on the SABM-BN curve, the new broad peak at 3431 cm^−1^ corresponds to the vibration of –OH [34]. For the products of saccharide-assisted ball-milled BN, extensive theoretical studies believe that H atoms in saccharides are preferentially combined with N atoms on BN to form N–H bonds [30,35,36,37], and the N–H bond on BN is easily hydrolyzed to generate NH_3_ and –OH. For this reason, the slightly alkaline pH of the BN-sucrose suspension (Figure 2c) can better verify the sucrose modification. Functionalized BN will improve the adhesion between the filler and the polymer matrix.

The SABM-BN contains exfoliating BNNS and larger size BN considering the ball milling efficiency. Therefore, the suspension is ultrasonically bathed (50 W) for 30 min before filtration and washing. Then the suspension is centrifuged at 2000 rpm for 30 min to separate the precipitate and supernatant, followed by filtration, washing, and drying to obtain BNTS and BNNS, respectively. The yields of BNTS and BNNS are 75.98% and 21.18%, respectively. However, the yield of BNNS is 10.24% without adding a grinding agent (sucrose). Although under high shear forces and collisions with the balls, the self-lubricating effect of BN sheets greatly reduces the exfoliation efficiency [38]. The addition of sucrose promotes the crushing of the sheets. The SEM images of BN, BNTS, and BNNS are shown in Figure 2b. The obtained BNNS has a Tyndall effect in an aqueous solution (~1 mg/mL, Figure 1). At the same concentration, BNNS has better dispersion stability than BNTS (Figure 2d) because larger size BNTS is more easily settled by gravity. All the above indicate that sucrose has a good effect on the exfoliation and modification of BN.

### 3.1. Thermal Properties Analysis

**Figure 3:**
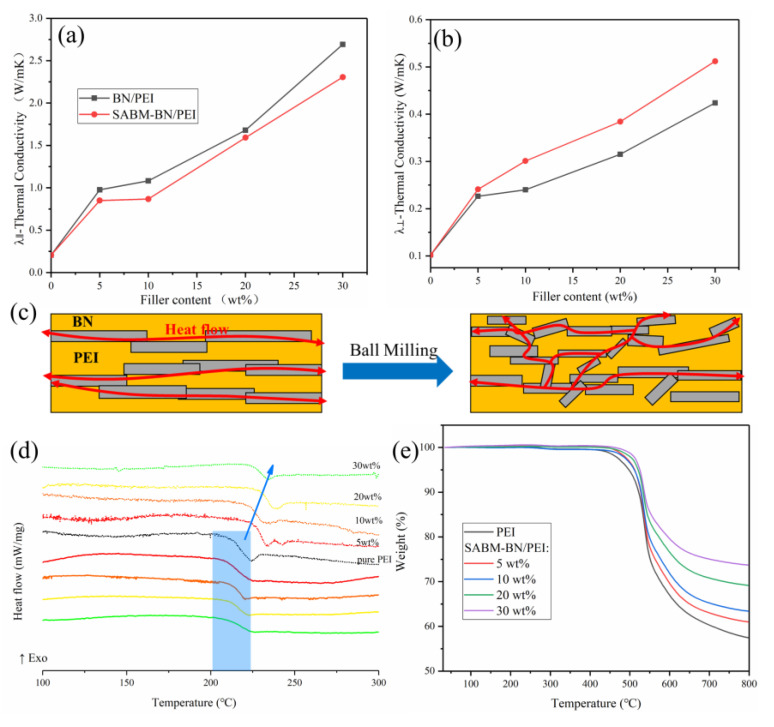
(**a**) In-plane and (**b**) through-plane thermal conductivity of PEI and the PEI composite films and (**c**) a heat conduction model. (**d**) DSC curves of PEI, the SABM-BN/PEI composite films (dashed line), and the pre-ball-milling BN/PEI composite film without sucrose (solid line). (**e**) TG curves of PEI and the SABM-BN/PEI composite films.

As mentioned above, the size of BN changed after ball milling. This will have an impact on the thermal conductivity of the composite film. Considering the anisotropy of film and sheet-like BN, the thermal conductivity of the composite film needs to be analyzed from both the horizontal and the vertical direction. Figure 3a shows the in-plane thermal conductivity (λ_∥_) of the PEI and composite films. The λ_∥_ of the 30 wt% BN/PEI composite film is 2.69 W/mK. The λ_∥_ of the SABM-BN/PEI composite film is lower than that of the BN/PEI composite film. The λ_∥_ of the 30 wt% SABM-BN/PEI composite film is 2.31 W/mK, which is ~11 times that of the pure film (0.205 W/mK). The through-plane thermal conductivity (λ_⊥_) of the PEI and composite films (Figure 3b) is much lower than that of the in-plane, which is owing to the orientation of molecular chain arrangement [39] and the low λ_⊥_ of BN (~10 W/mK) [32]. At the same time, it is found that λ_⊥_ of the SABM-BN/PEI composite film is improved. At 30 wt% content, the λ_⊥_ is 0.512 W/mK, higher than that of the BN/PEI composite film. We use the heat conduction model (Figure 3c) to explain. The large-sized BN tends to be arranged in parallel during the hot-pressing process. It is easier to form a thermal conduction network in the horizontal direction while the vertical direction is missing. However, after ball milling, the inconsistency of BN size destroys the continuity in the horizontal direction on the one hand. On the other hand, it gives the possibility to form a thermal network in the vertical direction.

Next, as shown in Figure 3d, the typical upper and lower tangent center is the glass transition temperature (Tg) of the polymer. The Tg of PEI is 215.6 ℃. The Tg of the SABM-BN/PEI composite films is higher than that of PEI. Furthermore, it is found that the Tg of the pre-ball-milling BN/PEI composite film without sucrose does not change significantly. It may be because the surface –OH of SABM-BN will also strengthen the interaction with polymer molecular chains, hindering the movements of PEI molecular chains and thereby increasing the Tg [40,41].

Under high power density, local heat accumulation in electronic devices and expansion for unique application scenarios, such as aerospace, military [10], etc., will place requirements on the heat resistance of the substrate. As an ether group-containing PI, PEI itself has excellent heat resistance. The TG curves of the composite films are shown in Figure 3e, with the accelerated decomposition of the films at ~550 ℃. The increasing residual rate with the increasing content indicates that the heat resistance of the composite films is further improved than that of the PEI film [42].

### 3.2. Application Assurance

Packaging substrates have excellent anti-voltage properties, which is the premise of ensuring the reliable operation of electronic devices. The anti-voltage properties of the composite films are tested by breakdown strength. The breakdown strength values of PEI and the PEI composite films are fitted by the Weibull distribution formula [43], as depicted in Equations (1) and (2).

(1) $$P=1-exp\left[-{\left(\frac{E_{i}}{E_{0}}\right)}^{\beta }\right]$$

(2) $$P=\frac{i-0.5}{n+0.25}$$

where *P* is the cumulative probability of electric failure, and *E_i_* is the *i*-th breakdown strength after the measured values are arranged from small to large. n represents the number of electrical breakdown points of the sample, and here are eight data points. *E_0_* is the Weibull breakdown strength at *p* = 63.2% under the linear fit of Equation (1). *β* is the shape distribution parameter, representing the discrete situation of the data.

**Figure 4:**
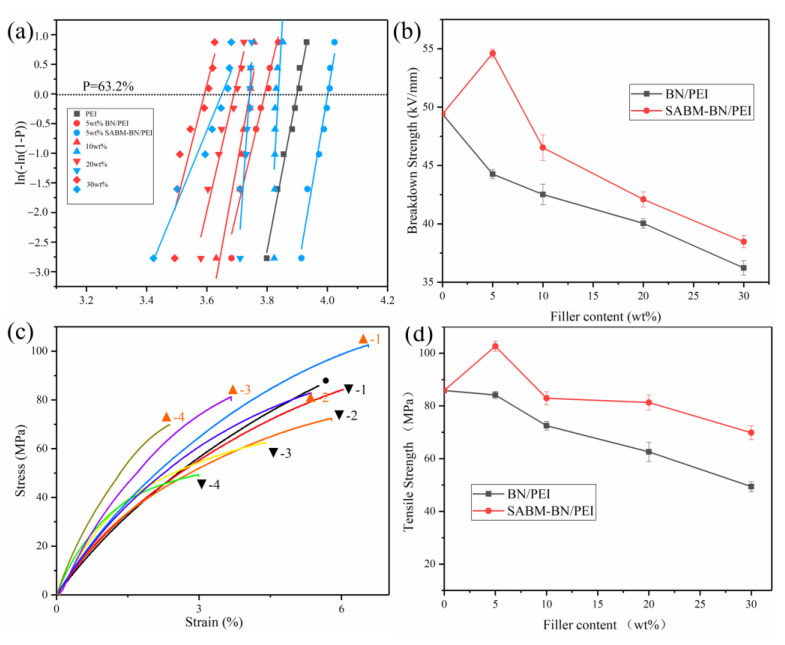
(**a**) Weibull distribution plots and (**b**) breakdown strength of PEI and the PEI composite films. (**c**) The stress-strain curves and (**d**) tensile strength of PEI and the PEI composite films. Black circle: PEI. Downward-facing black triangle 1–4: 5~30 wt% BN/PEI composite films. Upward-facing orange triangle 1–4: 5~30 wt% SABM-BN/PEI composite films.

Figure 4a,b show the Weibull distribution plots and breakdown strengths of PEI and the PEI composite films. As shown in Figure 4b, (i) the breakdown strength of the SABM-BN/PEI composite films is generally higher than that of the BN/PEI composite films, and the maximum value (54.6 kV/mm) appears when the content is 5 wt%. This is mainly owing to the contribution of BNNS. BNNS has an ultra-high theoretical withstand voltage performance (800 kV/mm). At the same time, after ball milling, the size reduction of BN will make the development of the electric tree during the breakdown process more tortuous [44]. Thus, the breakdown strength of the composite film is improved. (ii) With the increase of filler content, the breakdown strength of the films decreases from 49.4 kV/mm of PEI to 36.2 and 38.5 kV/mm. Since the increase of filler content will bring more defects, the generation of conductive paths will result [45]. Although the addition of high-content fillers reduces the breakdown strength of the composite film, it still meets the anti-voltage requirements in electronic devices (>5 kV/mm) [17].

Figure 4c shows stress-strain curves of PEI and the PEI composite films. The SABM-BN/PEI composite film curve is higher than that of the BN/PEI composite film at the same content. The tensile strength of the composite films is similar to the breakdown strength change (Figure 4d). The tensile strength of the PEI film is 85.87 MPa. The mechanical properties of the BN/PEI composite films decrease with the increasing content. At 30 wt% content, the tensile strength of the composite film is 49.38 MPa. The SABM-BN/PEI composite films have better mechanical properties than the BN/PEI composite films, and a typical “rise-fall” process occurs: the tensile strength of the 5 wt% SABM-BN/PEI composite film is 102.6 MPa, and at 30 wt% content, the tensile strength is 69.83 MPa. The improvement in tensile strength is attributed to the hydroxyl-rich surface of BN after ball milling, which promotes the compatibility of fillers with a polymer matrix and reduces stress concentration points [46].

### 3.3. Morphological Distribution

**Figure 5:**
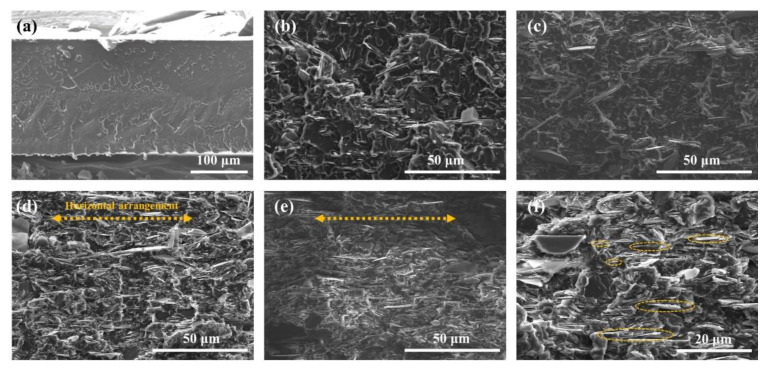
Cross-section SEM images of (**a**) PEI, (**b**) the 5 wt%, (**c**) 10 wt%, (**d**) 20 wt%, and (**e**) 30 wt% SABM-BN/PEI composite film. (**f**) Filler distribution at high magnification for the 20 wt% SABM-BN/PEI composite film.

Figure 5 shows the cross-section morphologies of PEI and the SABM-BN/PEI composite films. The cross-section of pure PEI film is denser than that of composite films. It is found that with the increase of filler content, the filler arrangement tends to be more horizontal. At 5 wt% content, the distribution of fillers has no obvious regularity, but at 20 wt% content, the distribution of fillers begins to tend to be horizontal. This is because the mutual volume between the fillers becomes more significant at high filler content so that it is distributed in parallel in the film under the external force of hot pressing [47]. It effectively explains the reason for the sudden increase in the in-plane thermal conductivity of the composite film at 20 wt% content; the heat conduction network in the horizontal direction has been formed. As shown in Figure 5f, the distribution of fillers of different sizes in the films is observed at high magnifications.

### 3.4. Thermal Management

**Figure 6:**
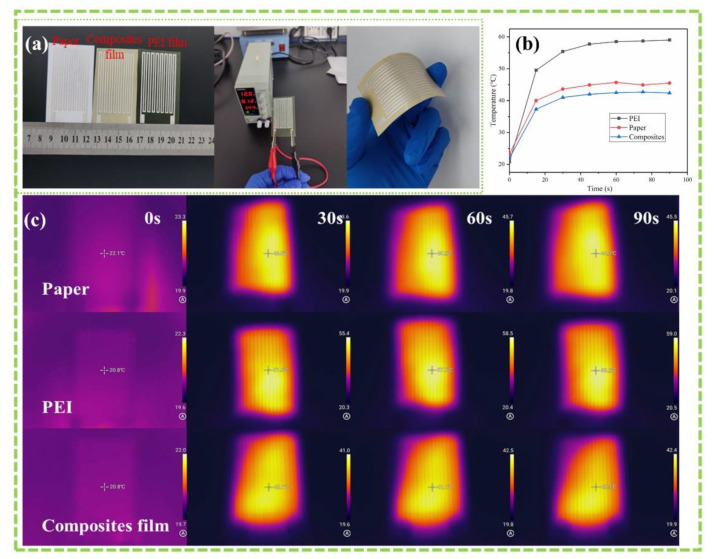
(**a**) From left to right: optical photographs of flexible circuits integrated into paper, PEI, and composite film; applying voltage; and bending the flexible substrate. (**b**) Surface temperature versus time. (**c**) Infrared thermal images.

To evaluate the thermal management ability of the prepared composite film as a flexible substrate, here we use screen printing to print circuits on the surface of the composite films (30 wt% SABM-BN/PEI). Paper and PEI film serve as controls. As shown in Figure 6a, the substrate size is tailored to 4 cm × 6.5 cm, the flexible circuit integrates with the PEI composite film that can be bent and has a certain flexibility, and then a constant voltage of 12 V is applied across the circuit. Owing to the existence of Joule heat, the film surface heats up immediately, and the surface heat distribution is shown in Figure 6c. It is found that the PEI film has a fast temperature rise rate, which is stable at around ~60 ℃, followed by the paper, which is stable at ~45 ℃. Importantly, the surface temperature of the PEI composite film was ~20 ℃ lower than that of the pure film, indicating that the “hot spot” temperature can be effectively reduced. Such a composite film substrate has excellent thermal management capability and can be used in flexible electronic devices with high energy density.

## 4. Conclusions

In summary, commercially available and cheap sucrose was chosen to exfoliate and modify BN simultaneously. The yield of BNNS in SABM-BN is ~21%, and SABM-BN has –OH groups. The size changes after ball milling and results in opposite differences in λ_∥_ and λ_⊥_ of the SABM-BN/PEI composite film and the BN/PEI composite film. The λ_∥_ of the SABM-BN/PEI composite film decreases to a certain extent compared with the BN/PEI composite film. At 30 wt% content, the BN/PEI composite film is 2.69 W/mK, while the SABM-BN/PEI composite film is 2.31 W/mK. However, the through-plane thermal conductivity of the SABM-BN/PEI composite film is improved. After ball-milling, the thermal conductive network reduces in the horizontal direction but becomes feasible in the vertical direction. Owing to the presence of BNNS and the modification of sucrose, the composite films’ breakdown strength and mechanical properties are improved. The maximum reaches 54.6 kV/mm and 102.7 MPa at 5 wt% content. Furthermore, as a flexible circuit substrate, the composite film has excellent thermal management capability. The working surface temperature is 20 °C lower than that of pure PEI film. We believe that this study provides an effective strategy for high-performance polymer composites for electronic packaging.

## Data Availability

Not applicable.
